# Zebrafish *prox1b* Mutants Develop a Lymphatic Vasculature, and *prox1b* Does Not Specifically Mark Lymphatic Endothelial Cells

**DOI:** 10.1371/journal.pone.0028934

**Published:** 2011-12-28

**Authors:** Shijie Tao, Merlijn Witte, Robert J. Bryson-Richardson, Peter D. Currie, Benjamin M. Hogan, Stefan Schulte-Merker

**Affiliations:** 1 Hubrecht Institute-Royal Netherlands Academy of Arts and Sciences and University Medical Centre, Utrecht, The Netherlands; 2 School of Biological Sciences, Monash University, Clayton, Victoria, Australia; 3 The Victor Chang Cardiac Research Institute, Darlinghurst, New South Wales, Australia; 4 St Vincent's Clinical School, Faculty of Medicine, University of New South Wales, New South Wales, Australia; 5 Australian Regenerative Medicine Institute, Monash University, Victoria, Australia; 6 EZO, Department of Animal Sciences, Wageningen University, Wageningen, The Netherlands; University of Birmingham, United Kingdom

## Abstract

**Background:**

The expression of the Prospero homeodomain transcription factor (Prox1) in a subset of cardinal venous cells specifies the lymphatic lineage in mice. Prox1 is also indispensible for the maintenance of lymphatic cell fate, and is therefore considered a master control gene for lymphangiogenesis in mammals. In zebrafish, there are two *prox1* paralogues, the previously described *prox1* (also known as *prox1a*) and the newly identified *prox1b*.

**Principal Findings:**

To investigate the role of the *prox1b* gene in zebrafish lymphangiogenesis, we knocked-down *prox1b* and found that depletion of *prox1b* mRNA did not cause lymphatic defects. We also generated two different *prox1b* mutant alleles, and maternal-zygotic homozygous mutant embryos were viable and did not show any lymphatic defects. Furthermore, the expression of *prox1b* was not restricted to lymphatic vessels during zebrafish development.

**Conclusion:**

We conclude that Prox1b activity is not essential for embryonic lymphatic development in zebrafish.

## Introduction

In vertebrates, in addition to the blood vasculature, the lymphatic vasculature plays important roles in maintaining an effective circulation. It is responsible for returning the protein-rich interstitial lymph fluid to the blood stream. Lymphatic vessels also contribute to the immune system that protects the body against infectious agents, and absorb lipids from the intestinal tract. Furthermore, there are a large number of inherited or acquired diseases that are associated with lymphatic vessel malfunction and lymphatic vessels may provide a primary route for the metastatic spread of certain tumors [1], [2], [3].

The lymphatic vessels were first described in 1627 by Aselli [4]. In 1902, Sabin proposed the origin of the lymphatic system from the veins [5]. The recent identification of several transcription factors, Prox1, Sox18, and CoupTF-II [6], [7], [8], which are essential for the specification of lymphatic cell fate, now provides a better understanding of the molecular mechanism of lymphatic development in metazoans.

In mice, the lymphatic system arises from a subset of venous endothelial cells (ECs) which start to express the homeodomain gene *Prox1* from E9.75. By subsequent polarized budding and sprouting, *Prox1*-expressing cells migrate away from the cardinal vein and give rise to the entire lymphatic system [6], [9]. In Prox1 null mice, lymphatic development is arrested, while the development of the blood vascular system is largely unaffected [6].

It has been reported that Prox1 plays similar roles in other vertebrate organisms, such as *Xenopus* and zebrafish [10], [11]. In zebrafish, the initial process of vasculogenesis leads to the formation of a primitive circulatory loop by 26 hpf (hours post fertilization), which consists of the posterior cardinal vein (PCV) and dorsal aorta (DA). A primary wave of angiogenesis, emanating from the DA, produces intersegmental arteries (ISAs), while a secondary wave of venous angiogenesis gives rise to sprouts that either remodel half of the existing ISAs into ISVs (intersegmental veins) by 2.5 dpf, or become parachordal lymphangioblasts (PLs), constituting a precursor pool of lymphangioblasts formed around 50 hpf at the horizontal myoseptum [12], [13]. At 60 hpf, the PLs start to migrate along ISAs, and move dorsally or ventrally to form the dorsal longitudinal lymphatic vessel (DLLV), the thoracic duct (TD) and the inter-segmental lymphatic vessels (ISLVs) that connect the DLLV and TD [14]. The presence of the TD is a commonly used indicator for normal lymphatic development. The *prox1b* gene was recently identified in zebrafish and its activity was claimed to be essential for zebrafish lymphangiogenesis and TD formation, using a morpholino knock-down approach [15]. Furthermore, *prox1b* was suggested to provide a molecular marker for lymphatic endothelial cells. We had performed similar experiments in the past, with different results. Here, we show that two mutant alleles of *prox1b* do not alter lymphatic development, and that *prox1b* is not a useful marker for lymphatic endothelial cells in zebrafish.

## Results

### *Prox1b* is expressed in endothelial cells and the cranial central nervous system

**Figure 1:**
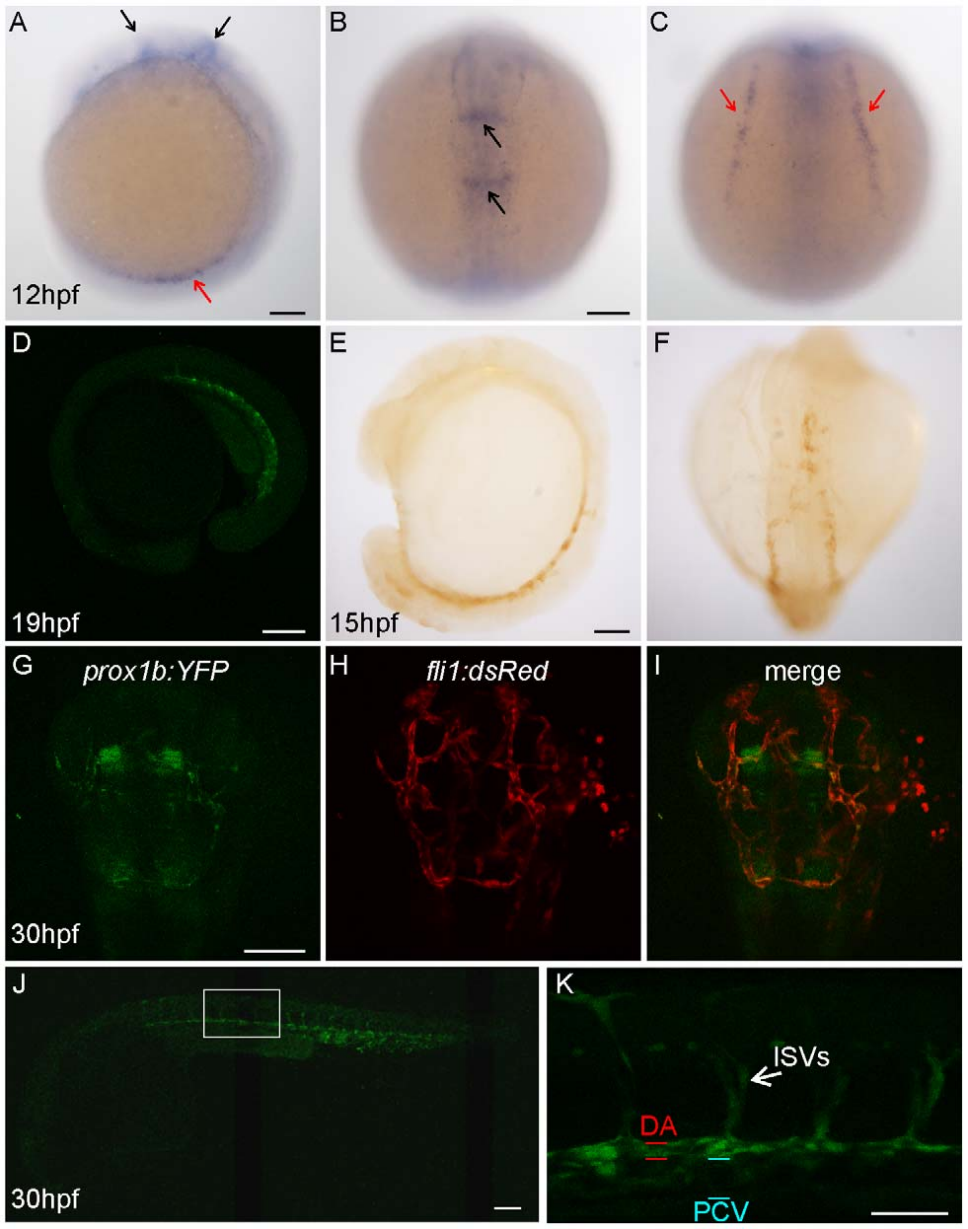
*Prox1b* is expressed in the endothelial cells and the central nervous system of the head. (A–C) shows *prox1b* transcript expression by whole mount *in situ* hybridization in wild-type embryos, at 12 hpf. Black arrows point to *prox1b* expression in the head; red arrows indicate *prox1b* expression in lateral plate mesoderm. Confocal image (D) shows YFP expression in a *prox1b BAC:YFP* embryo at 19 hpf stage. (E) and (F) show YFP expression, enhanced by DAB immunostaining, is detected in *prox1b BAC:YFP* embryos in migrating angioblasts at 15 hpf. (G–I) shows *prox1b:YFP* expression in the head region of a *prox1b BAC:YFP*, *fli1:DsRed* embryo. Note overlapping (endothelial cells) and non-overlapping expression domains. (J) shows *prox1b:YFP* expression in the trunk vasculature. (K) shows enlarged view of the boxed area in (J). Scale bars represent 50 µm in (K), and 100 µm in other figures.

**Figure 2:**
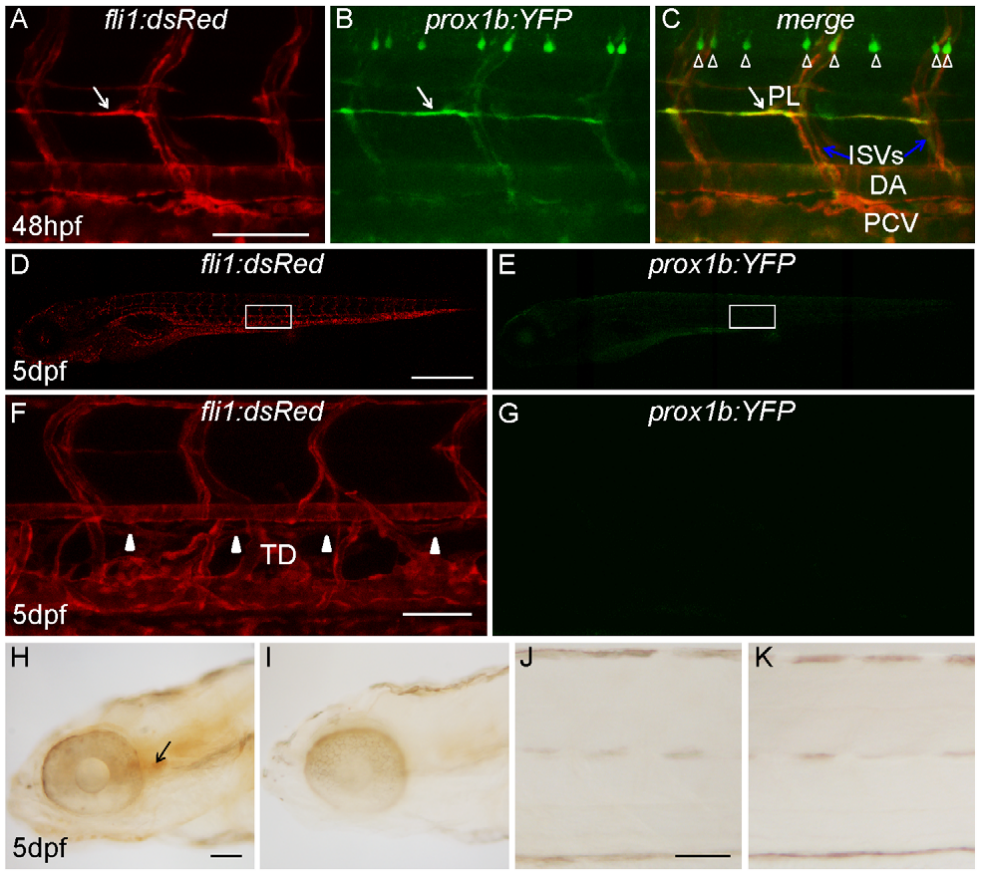
*Prox1b* does not specifically mark lymphatic aspects of the vasculature. (A–C) shows *prox1b:YFP* expression in motor neurons and all endothelial cells of a *prox1b BAC:YFP*, *fli1:DsRed* embryo at 48 hpf. White arrows point to parachordal lymphangioblasts. The white open arrowheads label motor neurons. Note that while there is expression of *prox1:YFP* in PLs, *prox1b* is also expressed in other (non-lymphatic) aspects of the vasculature. (D) and (E) show the fluorescence images of the same 5-day *prox1b BAC:YFP*, *fli1:DsRed* embryo. There is no detectable *prox1b:YFP* expression in the trunk region of the transgenic embryos (E). (F) and (G) show the enlarged views of the boxed area in (D) and (E). (F) White arrowheads indicate the TD, which resides between DA and PCV. (G) *prox1b:YFP* expression cannot be detected in TD. (H) and (J) show weak DAB immunostaining against YFP expression in the head (H, indicated by the black arrow), but not in the trunk of transgenic embryos (J). (I) and (K) are DAB staining controls without primary antibody. Scale bars represent 250 µm in (D), and 50 µm in other figures.

In mice, Prox1 is expressed in a variety of tissues other than lymphatic endothelial cells, including the lens, the heart, liver, pancreas, and central nervous system (CNS) [16]. By whole mount *in situ* hybridization, we were able to detect *prox1b* expression in the lateral plate mesoderm (LPM) and in the CNS of the head at 12 hpf stage (Figure 1 A–C). Since zebrafish endothelial cells (which provide the inner lining of blood and lymphatic vessels) originate from the LPM, we intended to follow *prox1b* over time. To that end, and in order to conveniently visualize the dynamic expression of *prox1b* gene in live embryo, we generated a Tol2-mediated bacterial artificial chromosome (BAC) transgenic line in zebrafish [17]. The overall morphology of the transgenic embryos appeared normal and was indistinguishable from wild type embryos (Figure 1D and J, and Figure 2D and E).

The *prox1b BAC:YFP* construct was created by inserting the yellow fluorescence protein (YFP) coding sequences at the position of the first translated ATG in a *prox1b*-harboring BAC (CH73-247L15) through homologous recombination [17]. This BAC contains approximately a 116-kb long zebrafish genomic contig, spanning from ∼97.7-kb upstream of the *prox1b* start codon to ∼18.6-kb downstream of the start codon, and harbors the entire *prox1b* coding sequence. We verified the correct knock-in of YFP in the *prox1b* gene based on diagnostic PCR (data not shown) and performed extensive analyses of the YFP expression in the *prox1b:YFP* embryos.

YFP expression in live embryo was not detected via confocal microscopy until late somitogenesis stages, when it was evident in the intermediate cell mass (derived from the earlier LPM and containing the trunk vascular precursors), but not in the head region (Figure 1D). To amplify the signal, we applied anti-GFP immunostaining with diaminobenzidin (DAB) solution in fixed embryos and were able to detect YFP expression in LPM as early as 15hpf (Figure 1E and F). Using standard *in situ* hybridization (ISH), we could confirm that *prox1b* is expressed, at 24 hpf, in the caudal vein (Figure S1A–C). As the development of the embryos proceeded, the expression level of YFP increased and signals also became apparent in more anterior aspects of the embryos (Figure 1G–I). At 30 hpf, in the head of *prox1b BAC*:YFP; *fli1:DsRed* double transgenic embryos [13], YFP expression partially overlapped with the red signals which marked all endothelial cells (Figure 1G–I). This indicates that the *prox1b* gene is expressed in both a subset of endothelial cells and some non-endothelial aspects of the central nervous system of the head. At the same stage, we detected bright YFP expression in all trunk endothelial cells: posterior cardinal vein (PCV), dorsal aorta (DA), and inter-segmental vessels (ISVs) (Figure 1J). In transgenic embryos we noted that the YFP signal level in the DA often appeared brighter than in the PCV (Figure 1K), however, using ISHs, we routinely observed that mRNA levels were equally high in the PCV (Figure S1D–F), sometimes even higher in the PCV than in the DA. Both YFP expression in transgenic embryos (Figure 2A–C) and ISHs confirm a rather broad expression in the caudal vein, the PCV and the DA. This dynamic *prox1b:YFP* expression in zebrafish ECs is hence different from *Prox1* expression in mice [6], which is, within endothelial cells, restricted to lymphatics, venous lymphatic precursors and venous valves [18].

### *Prox1b* expression is not restricted to lymphatic endothelial cells

In mice, Prox1 is required for both the specification of LEC fate and the maintenance of LEC cell identity. *Prox1* expression is not detected in the blood endothelial cells (BECs) after E11,5, but persists in the lymphatic vasculature until and throughout adulthood [18], [19]. We therefore examined the distribution of zebrafish *prox1b:YFP* expression and *prox1b* mRNA expression in late stage LECs. At 5 dpf, when the thoracic duct and intersegmental lymphatic vessels have formed, we were not able to detect any YFP expression in the trunk of *prox1b:YFP* embryos (Figure 2D–G and J, K), but observed weak signals in the head (Figure 2H and I).

*prox1b* ISH signals in the trunk of embryos at 48 hpf and 72 hpf had previously been reported [15]. Using a *prox1b* probe, we detected only diffuse expression in the trunk at these stages (Figure S1G–L), which we would normally consider non-specific. To test the specificity of this staining, we performed ISH for *prox1b* in *cloche* mutant embryos, which are devoid of all ECs (Figure S2A and B) [20], [21]. Since the staining pattern was unaltered in mutants, compared to wild-type embryos at 48 hpf and 72 hpf (Figure S2C, D and S1G, H, J, and K), we conclude that *prox1b* expression in ECs is no longer detectable by ISH from 48 hpf and after 72 hpf (Figure S1G–O). Therefore, different from *Prox1* expression in mice, *prox1*b is not a specific and persistent lineage marker for LECs in zebrafish.

### Knock-down of *prox1b* does not lead to impaired lymphatic development

As *prox1b* expression in the vasculature is not restricted to LECs, we wondered whether Prox1b is required for lymphatic development of zebrafish.

**Figure 3:**
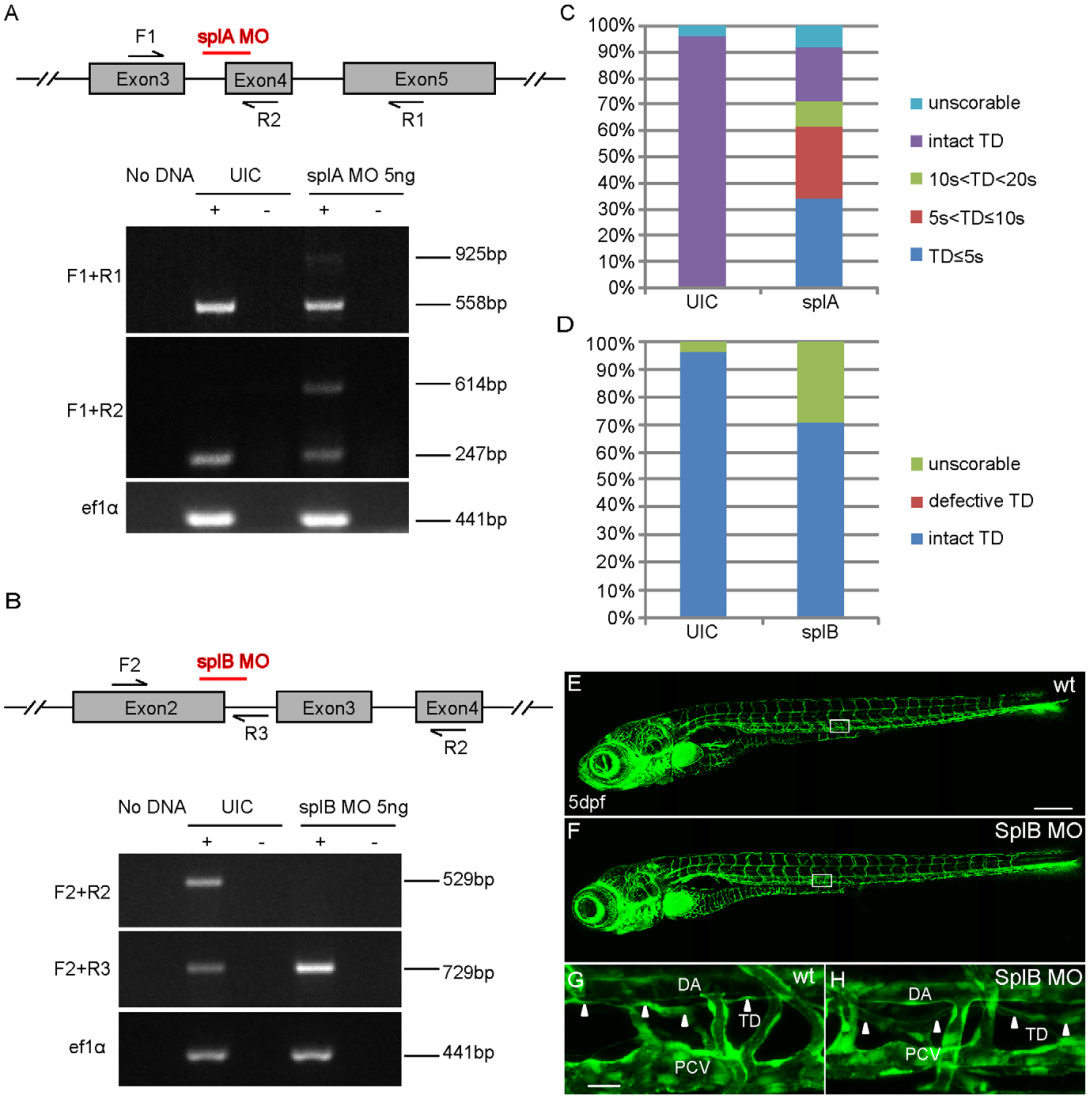
Morpholino-mediated knock-down of *prox1b* does not cause lymphatic phenotypes. (A) and (B) show schematics of the *prox1b* genomic locus with the red bars indicating target sites of the respective splice morpholinos. The numbered black arrows show the position of the primers used in RT-PCRs for examining the splicing of *prox1b* transcripts. (A) RT-PCR to detect splicing of *prox1b* in un-injected (UIC) and splA MO injected embryos. The expression of elongation factor 1-alpha (*ef1a*) gene represents the loading control. Primer pair F1 and R1 amplifies wild-type transcript band (558 bp) and incorrectly spliced transcripts (925 bp), which fail to excise the intron3-4 (367 bp). Primer pair F1 and R2 amplifies wild-type transcript (247 bp) and non-spliced transcripts (614 bp), which retain intron3-4. (B) RT-PCR to detect splicing of *prox1b* in un-injected and embryos injected with splB MO. Primer pair F2 and R2 amplifies wild-type transcripts (529 bp), which are missing in splB MO injected embryos. Primer pair F2 and R3 amplifies non-spliced transcripts (729 bp), which preferentially accumulated in morphant embryos. (C) Histograms showing the percentage of *fli1:GFP* embryos with different lengths of TD (10 s<TD<20 s means the partial TD covers the length of 10 to 20 somites in the trunk). Up to 70% of splA MO injected embryos displayed complete or partial loss of TD, even though splA MO seems not to affect *prox1b* splicing efficiently. Embryos were scored at 5 dpf. (D) Histograms showing the percentage of *fli1:GFP* embryos with intact or defective TD, and all the scorable embryos (their overall morphology was all right and they had normal blood circulation and did not develop edema at 5 dpf) developed complete TD after injection with splB MO. (E) and (F) show the full images of 5-day UIC (E) and splB MO injected embryos (F). (G) and (H) show enlarged views of the boxed areas in (E) and (F). The white arrowheads indicate the presence of TD in both control embryos (G) and morphants (H). Scale bars represent 250 µm in (E), and 25 µm in (G).

Initially, we used two different antisense morpholinos (splA MO and splB MO) to knock down *prox1b* gene activity. Both morpholinos were designed to target the splice sites of the *prox1b* gene (Figure 3A and B). The results of reverse transcription-PCR (RT-PCR) analysis showed that splA MO inefficiently blocked the excision of intron 3–4 (367 bp), leaving a substantial amount of correctly processed transcripts in morpholino injected embryos (Figure 3A), while injection of splB MO caused a loss of detectable wild-type *prox1b* transcripts, leading to the accumulation of non-spliced *prox1b* transcripts (retaining of intron2-3 leads to frame shift after aa494) (Figure 3B). However, ∼70% of splA MO injected embryos developed lymphatic defects: a complete or partial loss of TD at 5 dpf (Figure 3C). In contrast, we could not detect a similar phenotype in splB MO injected embryos (Figure 3D–H).

The presence of a TD in splB MO injected embryos indicates that loss of *prox1b* still allows for normal lymphatic development. The phenotypes observed in splA MO injected embryos are likely to be side-effects due to toxicity, and indeed we have in the past seen many examples where MO-injection affects TD-formation (unpublished observation). Since our data are in conflict with recently published information [15], we decided to generate stable mutant loss-of-function models, alleviating the need to have to rely on morpholinos.

### *Prox1b* mutants develop normally

We screened for *prox1b* mutants from an in-house TILLING (targeting induced local lesion in genome) library [22] and identified the *prox1b^hu3510^* allele (Figure S3A), with a stop mutation in the homeodomain of the gene. The mutant line was maintained in the *fli1:GFP* background, where all endothelial cells are marked by GFP expression [23], [24]. We observed that, at 48 hpf, the secondary venous sprouts occurred normally and the formation of PLs was unaffected in homozygous mutant embryos (Figure S3B and C). Indistinguishable from wild-type embryos, the homozygous mutants developed a completely normal TD at 5 dpf (Figure S3D–G). Homozygous *prox1b^hu3510^* mutant embryos were viable, although the mutation changed a highly conserved amino acid (W627) into a stop codon, which would be predicted to alter the function of the Prox1b Homeo-Prospero (HPD) domain by removing residues that bind the DNA phosphate backbone and by unmasking the C-terminal nuclear export sequence [25].

**Figure 4:**
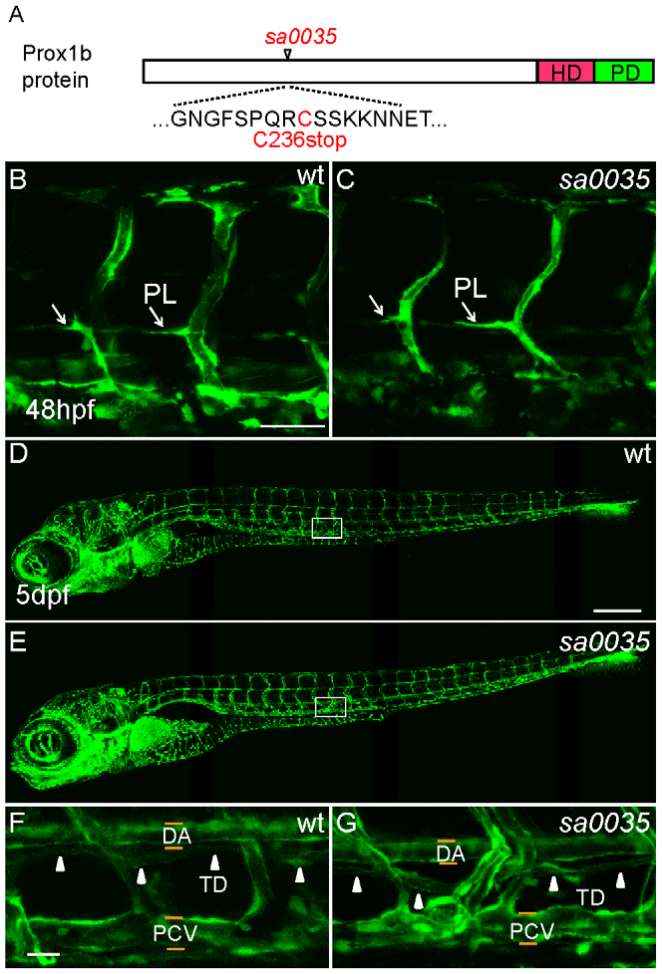
The lymphatic development of homozygous *prox1b^sa0035^* mutants appears normal. (A) Schematic representation of the Prox1b protein, with the position of the *prox1b^sa0035^* allele indicated. The homeodomain region (HD) is shown in red, the Prospero domain (PD) in green. The predicted stop mutation occurs at C236 in *prox1b^sa0035^*. (B) and (C) show vascular structures in the trunk region of wild-type (wt, B) and homozygous *prox1b^sa0035^* mutant embryos (C) in *fli1:GFP* background. The white arrows indicate PLs. (D) and (E) show whole embryo lateral view images of 5-day wt (D) and homozygous *prox1b^sa0035^* mutant embryos (E). (F) and (G) show enlarged views of the boxed areas in (D) and (E). The white arrowheads indicate the presence of TD in both control (F) and homozygous *prox1b^sa0035^* embryos (G). Scale bars represent 50 µm in (B), 250 µm in (D) and 25 µm in (F).

**Figure 5:**
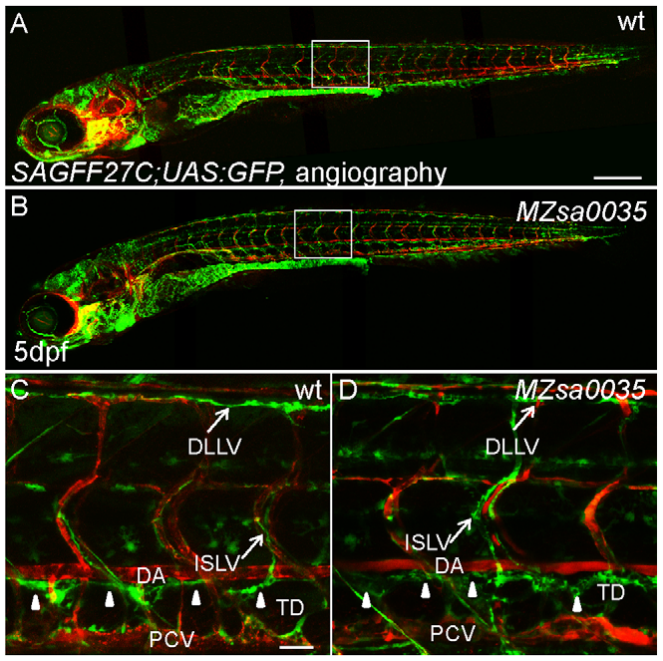
The lymphatic development of maternal-zygotic *prox1b^sa0035^* mutant is unaltered. (A) and (B) show whole embryo lateral view images of 5-day wt (A) and MZ *prox1b^sa0035^* mutant (B) in a *SAGFF27C;UAS:GFP* background. Perfused blood vessels were labeled by angiography (in red). (C) and (D) show enlarged views of the boxed areas in (A) and (B). The entire lymphatic network in the trunk of zebrafish, which is composed of the GFP-expressing lymphatic vessels-DLLV, ISLV and TD (marked by white arrowheads), is properly formed in wt (C) and MZ *prox1b^sa0035^* mutant embryos (D). Scale bars represent 250 µm in (A), and 25 µm in (C).

In an effort to obtain an additional allele, we received the *prox1b^sa0035^* line from the Sanger Centre. This allele harbors a mutation at amino acid 236. This mutation would predict a truncated Prox1 protein with the loss of the entire Homeo-Prospero domain, which is indispensable for Prox1 homologues to function as transcription factors (Figure 4A). We maintained the *prox1b^sa0035^* allele in *fli1:GFP* and *SAGFF27C;UAS:GFP* (heterozygous *SAGFF27C;UAS:GFP* line displays high reporter expression in lymphatic vasculature) transgenic background [12]. The *prox1b^sa0035^* homozygous mutant embryos were also viable and developed normally (Figure 4B–G). Both the specification and migration of the lymphatic endothelial cells were unaffected in the mutants, as the formation of PLs (Figure 4B and C) and TD was indistinguishable from wild type embryos (Figure 4F and G). The *prox1b^sa0035^* homozygous fish are fertile. To exclude the possibility that maternal *prox1b* mRNA might rescue a possible homozygous zygotic phenotype, we crossed *prox1b^sa0035−/−^, SAGFF27C;UAS:GFP* fish, and generated maternal-zygotic (MZ) *prox1b* mutant progeny. These MZ mutant embryos have no visible defects (Figure 5A and B) and we observed a properly formed embryonic lymphatic network, including DLLV, ISLVs and TD, at 5 dpf (Figure 5C and D).

The analysis of two *prox1b* mutant alleles demonstrates that *prox1b* gene activity is not necessary for any known indispensible functional process during the development of zebrafish. In combination with the *prox1b* expression and knock-down analysis, we conclude that Prox1b activity is not essential for zebrafish embryonic lymphatic development.

## Discussion

It has been recently reported that zebrafish *prox1b* expression marks lymphatic endothelial cells, and that knock-down of *prox1b* via morpholinos completely abolishes lymphangiogenesis [15]. We here report that two mutant alleles of *prox1b* undergo normal lymphangiogenesis, and that, at least at later stages of development, *prox1b* expression does not mark lymphatic endothelial cells.

In mammals, the activities of the transcription factors Coup-TFII [8], Sox 18 [7] and Prox1 [6] lead to the specification of a subpopulation of anterior cardinal vein cells into future lymphatic endothelial cells (LECs) [6], [7], [8]. Subsequently, Ccbe1, VEGF-C and its receptor, VEGFR3, are required for outgrowth of these prospective LECs. For the latter process, there is excellent genetic evidence for strong evolutionary conservation between mice and zebrafish: mutations in Ccbe1 [14], [26], VegfC and Vegfr3 [11], [27], [28], [29] are deficient in most aspects of venous sprouting and consequently lack a thoracic duct. The phenotypic effects of lacking Coup-TFII, Sox 18 and Prox1 are less well supported in zebrafish (no mutants for either of these genes have been reported) and have started to be elucidated only recently. Knock-down of Coup-TFII results in lymphatic defects [30], but lack of Sox18 has been independently reported to have no phenotype by three groups [31], [32], [33]. Since the effect of Sox18 in mice is context-dependent, this might not be completely surprising [7]. Finally, in the case of Prox1, a recent study reported a requirement for *prox1b* in embryonic lymphatic formation [15].

Our conclusion that Prox1b activity is not essential for lymphatic development, is contradictory to a previous report [15]. In previous work, the function of Prox1b was studied by using a morpholino knock-down approach. Only a single morpholino covering the first translated ATG was employed in the study, however, the authors demonstrated rescue of the morphant phenotype by injection of mRNA. Here, we show analysis with a splice (splB) morpholino, which we demonstrate to deplete nearly all wild-type *prox1b* transcripts, while not causing any lymphatic defects. The presence of a TD in splB MO injected embryos indicates that loss of *prox1b* still allows normal lymphatic development. Although we cannot explain the published mRNA rescue experiments, all our results indicate that the lymphatic defects observed in ATG and splA MO injected embryos are most likely not due to loss of Prox1b function. Of note, the *prox1b* morpholino used in the previous study causes severe edema at 4 dpf. We take this as evidence of considerable toxicity of the morpholino, as from previous studies [14], [29], it is clear that lack of lymphatic tissue does not cause edema at this early stage. The toxicity might be the cause for the reduction of *lyve-1* expression in morphants.

Furthermore, we analyzed two mutant alleles that provide an additional method to study *prox1b* function. Homozygous mutants for both alleles, and even maternal-zygotic mutants, develop normally without visible defects. Prox1b shares high homology with Drosophila Prospero (Pros) in the homeodomain and prospero domain (PD). Both proteins contain a nuclear export signal (NES) in the homeodomain. Based on a previous study [25], a physiological signal induces Pros to adopt a closed conformation so that the PD shields the NES and Pros enters the nucleus and is prepared for DNA binding. If the NES masking is turned off or blocked, Pros adopts an open structure that exposes NES and exits the nucleus [25]. The *prox1b^hu3510^* allele harbors a non-sense mutation, which deletes the C-terminal part of the PD, which contains a highly conserved DNA binding residue Lys629, and is also essential to physically cover the NES in the homeodomain, so the predicted truncated protein is unlikely to adopt the closed conformation and be kept in the nucleus for proper DNA binding.

The other allele, *prox1b^sa0035^*, is predicted to be truncated after the coil-coil domain, and does not contain the homeodomain and prospero domain. Therefore, we believe that both mutants almost certainly lack wild-type activity of Prox1b, and that at least the *prox1b^sa0035^* allele represents a loss-of-function situation. Although we cannot exclude the possibility of a compensatory transcriptional mechanism involving other *prox* homologues, or of a complex transcript processing event to remove these coding mutations, we have not been able to detect any evidence to support either of these hypotheses. Injections of *prox1a* morpholinos into *prox1b* mutants at lower dose did not affect TD formation (data not shown; low doses of morpholinos had to be used since *prox1a* morpholinos are toxic). Based on two mutant alleles and the splB morpholino, we conclude that the activity of Prox1b is dispensable during zebrafish development.

In zebrafish, LECs arise from the PCV sprouts that do not connect to ISAs, and approximately half of the venous spouts fall into this class. These sprouts migrate to the horizontal midline and become lymphatic precursors, dubbed PLs [14]. It is unclear at present whether the lymphatic fate of these venous sprouts is determined before they emanate from the PCV, or whether they get specified during their migration from PCV to horizontal myoseptum. If *prox1b* was a specific LEC lineage marker comparable to mammalian *Prox*1, its early expression around 30 hpf, would be expected to be found in a polarized manner, present in a salt-and-pepper pattern in the dorsal side of the PCV or restricted to half of the venous spouts that constitute future PLs. At later stages, *prox1b* expression should only be observed in lymphatic endothelial cells (PLs and TD, DLLV, and ISLVs) but not blood endothelial cells. This is not what we observe: rather, *prox1b* expression is detected in all endothelial cells and is not enriched at the dorsal side of the PCV around 30 hpf. After that point in time, *prox1b* expression in endothelial cells becomes increasingly weaker. Although a few PLs seem to have brighter expression at 48 hpf, *prox1b* expression is never exclusively observed in LECs, instead it is also present in BECs (Figure 2A–C). Furthermore, *prox1b* expression is not maintained in LECs, and eventually it completely disappears from the zebrafish trunk vasculature, and at 5 days there was no expression detectable in the TD or any other trunk endothelial tissue (Figure 2G, J and S1M–O). Based on our results, *prox1b* is not a LEC-specific marker in zebrafish.

In mice, the expression of Coup-TFII in the blood vasculature is restricted to veins and it inhibits Notch activity and blocks an arterial signaling cascade in venous endothelial cells (VECs) [34]. At embryonic day E9, Sox18 expression becomes apparent in a subset of cells in the anterior cardinal vein and induces *Prox1* expression in these cells around E9.75 [7], [35], in synergy with Coup-TFII [8]. The direct interaction between Coup-TFII and Prox1 is also necessary for the maintenance of *Prox1* expression during early stages of LEC specification and differentiation [8], [36], [37]. In zebrafish, the lymphatic cells also arise from VECs, the secondary sprouts of the PCV [12], [13]. However, the molecular mechanisms that initiate lymphatic specification remain unclear and might be different from mammals. Recent work from Aranguren et al., indicates a conserved role of Coup-TFII for venous and lymphatic development in zebrafish [30]. Knockdown of Sox18 alone failed to reveal any phenotype [32], [33], and the simultaneous knockdown of sox18 and sox7 severely affected the arteriovenous identity and led to dramatic fusion between the major axial vessels [31], [32]. So far, there is no clear evidence to show that SoxF proteins have a role in zebrafish lymphatic development; however, in mice the function of Sox18 in early aspects of lymphangiogenesis is also context dependent [7]. It remains possible that a homeodomain transcription factor, perhaps a Prox homologue, functions directly downstream of Coup-TFII to switch on the lymphatic lineage in zebrafish. Since we demonstrate here that Prox1b is not required for normal lymphatic development, Prox1a becomes the most likely candidate. It was previously reported that the morpholinos targeting zebrafish *prox1a* gene usually led to severely delayed or impaired growth at early stages and eventually caused massive malformations before a lymphatic phenotype could reasonably be scored [27]. Therefore, a yet to be identified *prox1a* mutant would be required to shed further light onto the molecular pathway that initiates the lymphatic fate in zebrafish.

## Methods

### Ethics statement

All zebrafish strains were maintained under standard husbandry conditions at the Hubrecht Institute, and animal experiments were approved by the Animal Experimentation Committee (DEC) of the Royal Netherlands Academy of Arts and Sciences. Permit number for this study is: 80101.

### Morpholinos

All morpholinos were obtained from GeneTools. *Prox1b* splA MO (5′-CACAGCGATTGAACTGTGTAGCGAG-3′) was designed to target the intron3-4/exon4 boundary, while *prox1b* splB MO (5′-GATAAAAGGATATTGAACCTGCAGC-3′) was designed to target the exon2/intron2-3 boundary. Both of the morpholinos were injected at a concentration of 5 ng/embryo. All morpholinos and primers were designed according to the *prox1b* sequence in GenBank/EMBL database under the accession number FJ544314.

### RT-PCR

Total RNA (1 µg) from wildtype and morpholino injected embryos was reverse transcribed by using M-MLV Reverse Transcriptase and random hexamers (Promega). PCR followed by sequencing was performed with the following primers:

F1: 5′-GCCACTTGAAGAAAGCCAAG-3′

F2: 5′-GCCCCTTCTTCACTACACCA-3′

R1: 5′-CCTCCAGAACCAGCAATAAG-3′

R2:5′-CGGTAAAGCTCGGTGTCTCT-3′

R3: 5′-GTGTGGTCCCTGTTGATCCT-3′.

### *In situ* hybridization

*In situ* hybridization was performed as previously described [38]. *Prox1b* probe was synthesized by in vitro transcription from the *HindIII*-digested full length cDNA in pBSK, using T7 RNA polymerase (Promega).

Embryos were subjected to ISH and paraffin sections (6 µm) were counter-stained with neutral red.

### Microangiography

We performed microangiography as described [27]. Embryos were anesthetized in 0.015% MS-222 at 5 dpf and were embedded in 0.5% low melting point agarose. A small bulb of tetramethylrhodamine (TAMRA; Molecular Probes, 2,000,000 MW, 10 mg/ml) was administered by cardiac injection using a conventional microinjection setup. Only embryos exhibiting TAMRA throughout the cardiovascular system immediately after the injection were further analyzed [27].

### Microscopy

Embryos were mounted in 0.5% low melting point agarose in a culture dish with a cover slip replacing the bottom. Imaging was performed with a Leica SPE confocal microscope using a 10× objective with digital zoom.

### Diaminobenzidine (DAB) histochemistry

DAB staining was performed using VECTASTAIN ABC kit (VECTOR) and DAB tablet (SIGMA). The expression of YFP was detected by using rabbit polyclonal anti-GFP (1∶500, Torrey Pines Biolabs, Acris TP401) as primary antibody and polyclonal swine anti-rabbit Ig/Biotinylated (1∶500, DAKO, E0353) as secondary antibody.

### Mutants

*prox1b^hu3510^* allele was identified from an ENU *mutagene*sis library in Hubrecht Institute by Tilling. The *prox1b^sa0035^* allele was ordered from Zebrafish Mutation Resource of Sanger Institute. Mutants were genotyped by using the following KASPAR primers:

*prox1b^hu3510^* ID1_ALG 5′-GAAGGTGACCAAGTTCATGCTCAGATGATCTTATAGATGGACTTCTTC-3′

*prox1b^hu3510^* ID2_ALA 5′-GAAGGTCGGAGTCAACGGATTACAGATGATCTTATAGATGGACTTCTTT-3′

*prox1b^hu3510^* ID-C1 5′-GCCATCCAGAGCGGTCGGGAT-3′

*prox1b^sa0035^* ID_ALT 5′-GAAGGTGACCAAGTTCATGCTGCAATGGATTTTCTCCTCAACGTTGT-3′

*prox1b^sa0035^* ID_ALA 5′-GAAGGTCGGAGTCAACGGATTGCAATGGATTTTCTCCTCAACGTTGA-3′

*prox1b^sa0035^* ID_C1 5′-GGAAAAATTCCAGCATTGCCATTTCCATT-3′

The *cloche^t22499^* allele has been previously described [32].

### Generation of transgenic line

Generation of transgenic lines with bacterial artificial chromosome (BAC) was described previously [17]. For the *prox1b BAC:YFP* transgenic line, a citrine-neomycin cassette was recombined by using Red/ET Recombination Technology (Gene Bridges), into the bacterial artificial chromosome (BAC) clones CH73-247L15 using homology arm tagged PCR primers. DNA was injected into one cell-stage embryos at a concentration of 500 pg/embryo. A transgenic carrier adult was selected by screening for fluorescent progeny.

## Supporting Information

Figure S1 ***Prox1b*** **transcript expression in zebrafish.** (A–O) shows *prox1b* expression analyzed by *in situ* hybridization in whole mount embryos (A, D, E, G, H, J, K, M and N) and transverse sections (B, C, F, I, L, and O), at different stages: 24 hpf (A–C), 32 hpf (D–F), 48 hpf (G–I), 72 hpf (J–L) and 5 dpf (M–O). (E), (H), (K) and (N) individually show the enlarged views of the boxed area in (D), (G), (J) and (M). The blue and red bars in (A) represent the positions of the sections in (B) and (C). *prox1b* expression is prominent in the caudal vein of embryos at 24 hpf (C) and in both the DA and PCV at 32 hpf stage, shown by (E) and (F). (G–O) However, there is no signal in the blood and lymphatic endothelial cells of older embryos at 48 hpf, 72 hpf and 5 dpf. The black arrows point to the dorsal aorta; the blue arrows point to the posterior cardinal vein or caudal vein; and the red arrows point to thoracic duct. The black and blue arrow heads point to the *prox1b* expression in the DA and PCV separately. NT: neural tube; NC: notochord. Scale bars represent 200 µm in (A), (D), (G), (J), (M); 50 µm in (E), (H), (K) and (N); and 20 µm in (B), (C), (F), (I), (L) and (O).
(TIF)

Figure S2 ***Prox1b*** **expression in** ***cloche*** **mutant.** (A–D) shows *prox1b* expression in *cloche* mutants and siblings. The red and blue arrows point to *prox1b* expression in the DA and PCV of a sibling embryo separately (A), while this *prox1b* expression is absent in *cloche* mutant (B). The black arrows point to signals in the ventral region of the trunk (C) and along the somite boundaries of homozygous *cloche* mutants (D). (E) The inset shows an enlarged view of the boxed area in (D). The staining indicated by black arrows in C, D and E is either non-endothelial or non-specific because *cloche* mutant embryos lack endothelial cells. Given the diffuse nature of the staining, the black arrow indicated signal is probably a non-specific artifact of over-staining. Scale bars represent 100 µm in (A), and 50 µm in (E).
(TIF)

Figure S3 **The lymphatic development of homozygous** ***prox1b^hu3510^*** **mutant is normal.** (A) Schematic representation of the Prox1b protein, with the position of the *prox1b^hu3510^* allele indicated. The homeodomain region is shown in red, the Prospero domain in green. The predicted stop mutation occurs at W627 in *prox1b^hu3510^* and multiple sequence alignment shows the conservation of zebrafish W627 in the Prospero domain of Prox proteins. (B) and (C) show the vascular structures in the trunk region of wt (B) and homozygous *prox1b^hu3510^* mutant embryos (C) in *fli1:GFP* background. The white arrows indicate PLs. (D) and (E) show the full images of 5-day wt (D) and homozygous *prox1b^hu3510^* mutant embryos (E). (F) and (G) show enlarged views of the boxed areas in (D) and (E). The white arrowheads indicate the presence of TD in both control (F) and homozygous *prox1b^hu3510^* embryos (G). Scale bars represent 50 µm in (B), 250 µm in (D) and 25 µm in (F).
(TIF)
