# Supplementary figures and images for: Zebrafish prox1b Mutants Develop a Lymphatic Vasculature, and prox1b Does Not Specifically Mark Lymphatic Endothelial Cells
Source: PLoS One. 2011 Dec 28;6(12):e28934. doi: 10.1371/journal.pone.0028934 (PMC3247213; doi:10.1371/journal.pone.0028934)

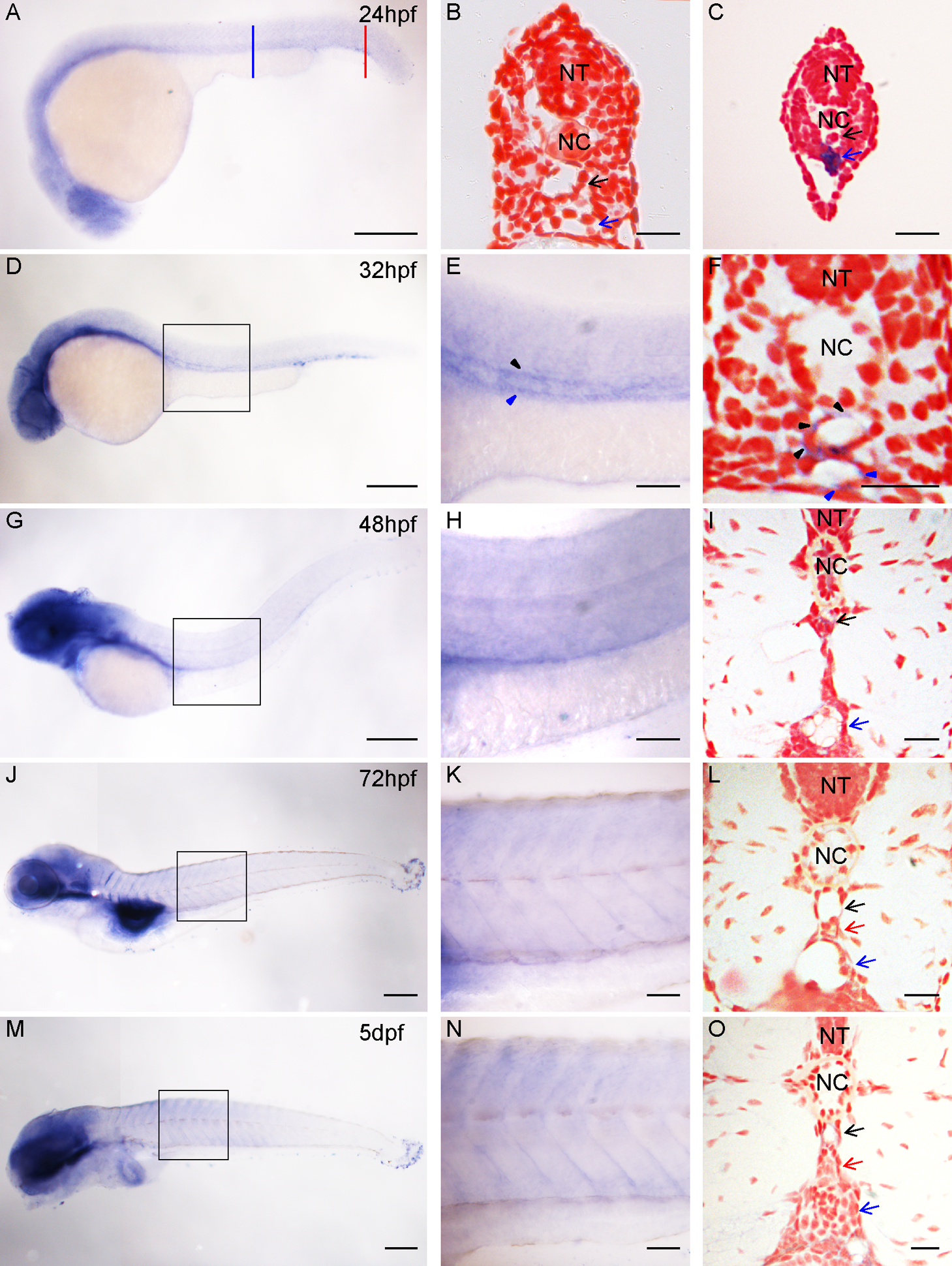

Supplement: Figure S1 — Prox1b transcript expression in zebrafish. (A–O) shows prox1b expression analyzed by in situ hybridization in whole mount embryos (A, D, E, G, H, J, K, M and N) and transverse sections (B, C, F, I, L, and O), at different stages: 24 hpf (A–C), 32 hpf (D–F), 48 hpf (G–I), 72 hpf (J–L) and 5 dpf (M–O). (E), (H), (K) and (N) individually show the enlarged views of the boxed area in (D), (G), (J) and (M). The blue and red bars in (A) represent the positions of the sections in (B) and (C). prox1b expression is prominent in the caudal vein of embryos at 24 hpf (C) and in both the DA and PCV at 32 hpf stage, shown by (E) and (F). (G–O) However, there is no signal in the blood and lymphatic endothelial cells of older embryos at 48 hpf, 72 hpf and 5 dpf. The black arrows point to the dorsal aorta; the blue arrows point to the posterior cardinal vein or caudal vein; and the red arrows point to thoracic duct. The black and blue arrow heads point to the prox1b expression in the DA and PCV separately. NT: neural tube; NC: notochord. Scale bars represent 200 µm in (A), (D), (G), (J), (M); 50 µm in (E), (H), (K) and (N); and 20 µm in (B), (C), (F), (I), (L) and (O). (TIF) [file pone.0028934.s001.tif]

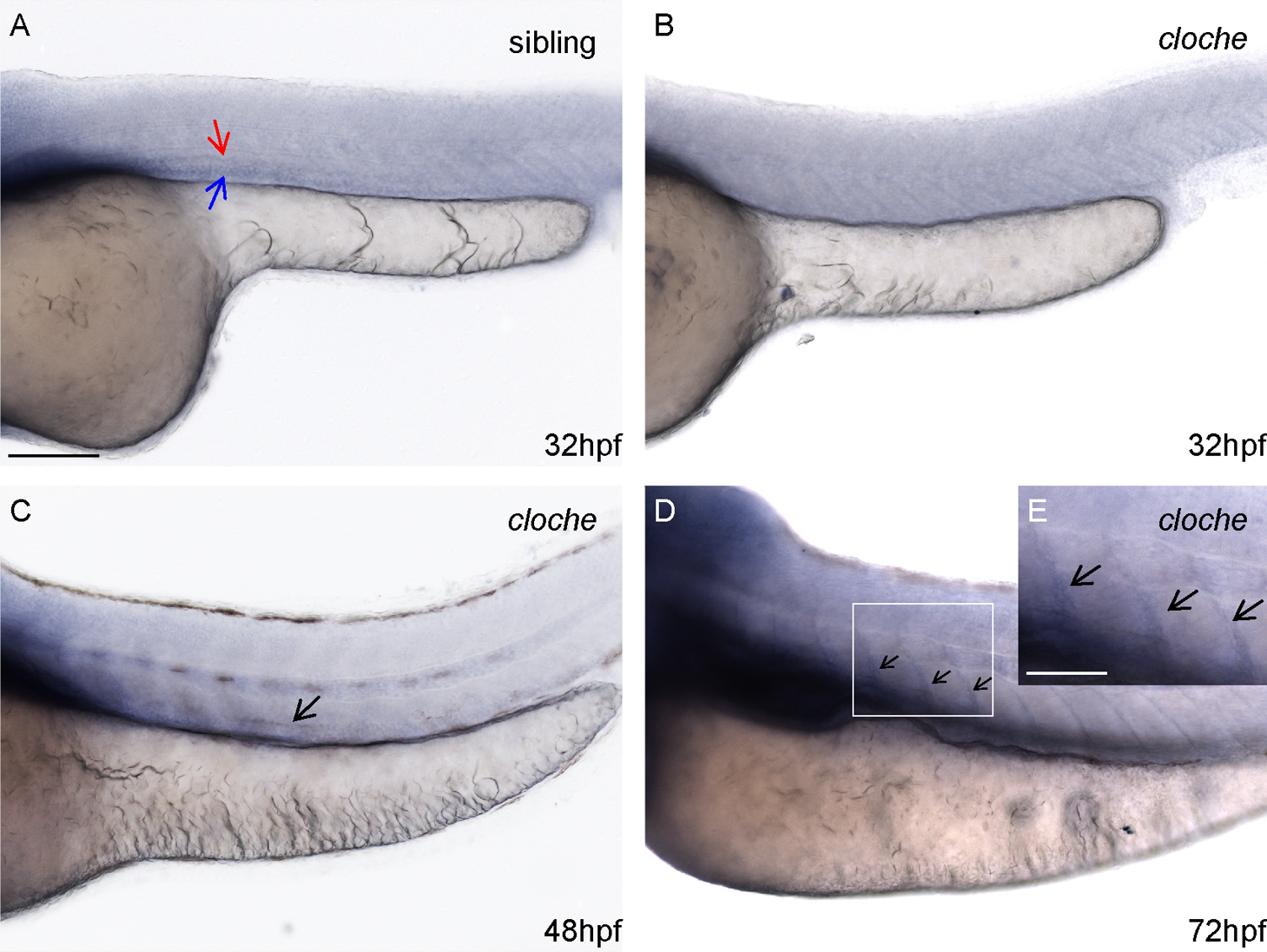

Supplement: Figure S2 — Prox1b expression in cloche mutant. (A–D) shows prox1b expression in cloche mutants and siblings. The red and blue arrows point to prox1b expression in the DA and PCV of a sibling embryo separately (A), while this prox1b expression is absent in cloche mutant (B). The black arrows point to signals in the ventral region of the trunk (C) and along the somite boundaries of homozygous cloche mutants (D). (E) The inset shows an enlarged view of the boxed area in (D). The staining indicated by black arrows in C, D and E is either non-endothelial or non-specific because cloche mutant embryos lack endothelial cells. Given the diffuse nature of the staining, the black arrow indicated signal is probably a non-specific artifact of over-staining. Scale bars represent 100 µm in (A), and 50 µm in (E). (TIF) [file pone.0028934.s002.tif]

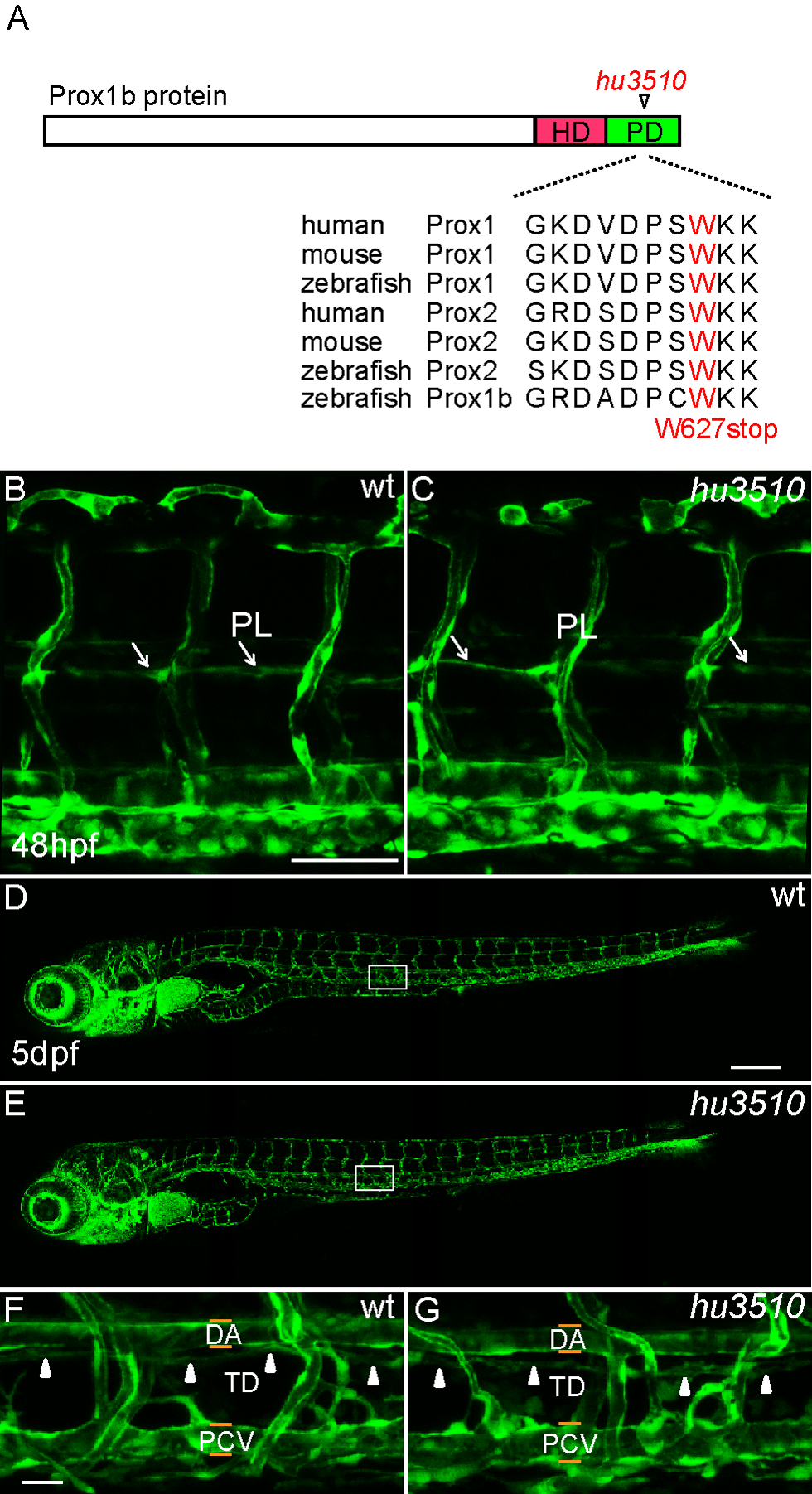

Supplement: Figure S3 — The lymphatic development of homozygous prox1bhu3510 mutant is normal. (A) Schematic representation of the Prox1b protein, with the position of the prox1bhu3510 allele indicated. The homeodomain region is shown in red, the Prospero domain in green. The predicted stop mutation occurs at W627 in prox1bhu3510 and multiple sequence alignment shows the conservation of zebrafish W627 in the Prospero domain of Prox proteins. (B) and (C) show the vascular structures in the trunk region of wt (B) and homozygous prox1bhu3510 mutant embryos (C) in fli1:GFP background. The white arrows indicate PLs. (D) and (E) show the full images of 5-day wt (D) and homozygous prox1bhu3510 mutant embryos (E). (F) and (G) show enlarged views of the boxed areas in (D) and (E). The white arrowheads indicate the presence of TD in both control (F) and homozygous prox1bhu3510 embryos (G). Scale bars represent 50 µm in (B), 250 µm in (D) and 25 µm in (F). (TIF) [file pone.0028934.s003.tif]
